# Antibody Specific B-Cell Epitope Predictions: Leveraging Information From Antibody-Antigen Protein Complexes

**DOI:** 10.3389/fimmu.2019.00298

**Published:** 2019-02-26

**Authors:** Martin Closter Jespersen, Swapnil Mahajan, Bjoern Peters, Morten Nielsen, Paolo Marcatili

**Affiliations:** ^1^Department of Bio and Health Informatics, Technical University of Denmark, Kongens Lyngby, Denmark; ^2^La Jolla Institute for Allergy and Immunology, Center for Infectious Disease, Allergy and Asthma Research, La Jolla, CA, United States; ^3^Instituto de Investigaciones Biotecnológicas, Universidad Nacional de San Martín, Buenos Aires, Argentina

**Keywords:** antigen, antibody, B cell epitope, prediction, paratope, antibody specific epitope prediction

## Abstract

B-cells can neutralize pathogenic molecules by targeting them with extreme specificity using receptors secreted or expressed on their surface (antibodies). This is achieved via molecular interactions between the paratope (i.e., the antibody residues involved in the binding) and the interacting region (epitope) of its target molecule (antigen). Discerning the rules that define this specificity would have profound implications for our understanding of humoral immunogenicity and its applications. The aim of this work is to produce improved, antibody-specific epitope predictions by exploiting features derived from the antigens and their cognate antibodies structures, and combining them using statistical and machine learning algorithms. We have identified several geometric and physicochemical features that are correlated in interacting paratopes and epitopes, used them to develop a Monte Carlo algorithm to generate putative epitopes-paratope pairs, and train a machine-learning model to score them. We show that, by including the structural and physicochemical properties of the paratope, we improve the prediction of the target of a given B-cell receptor. Moreover, we demonstrate a gain in predictive power both in terms of identifying the cognate antigen target for a given antibody and the antibody target for a given antigen, exceeding the results of other available tools.

## Introduction

B-cells form an essential part of the adaptive immune system, as they are capable of providing long-term protection against pathogens and harmful molecules. Their extremely specific B-cell receptors, named immunoglobulins or antibodies, are key components in this process. Antibodies recognize their molecular targets, termed antigens, via interactions between their binding site (paratope) and a specific region of the antigen (epitope).

Most B-cell epitopes are discontinuous in sequence, meaning that they are composed of residues that might be far apart in sequence and are brought together in spatial proximity by the protein folding (1). Data describing such conformational epitopes are mainly obtained from experimentally resolved 3D structures of antibodies co-crystallized with their target antigen, which allows a very precise identification of the epitope residues.

Identification of B-cell epitopes is of high importance for many medical, immunological and biological applications including disease control, diagnostics, and vaccine development (2). Several experimental methods for epitope identification are available including protein crystallography, ELISA and peptide-chip, but in general they are expensive, time consuming, low-throughput, or have low accuracy. Several computational methods have been developed to assist or substitute the experimental approaches, including BepiPred, DiscoTope, CBtope, and ABCpred (3–6). In absence of information on the cognate antibody, B-cell epitope prediction tools can broadly be categorized in two groups: sequence- and structure-based methods. As the names suggest, sequence-based methods predict the B-cell epitopes from the protein sequence of the antigen alone, whereas structure-based methods take into account also their 3D structure. Many benchmark studies have, as expected, demonstrated that structure-based methods display superior performance compared to sequence-based methods (7). However, even the best current structure-based methods for B-cell epitope prediction have limited predictive power (4). One important reason for this is that in many cases the problem is ill-posed. If we set the task in very broad terms, we aim at predicting whether a given surface patch of an antigen is a potential epitope, i.e., if one or more of the many billion antibodies potentially present in a host can target this region. Formulated like this, most surface patches of an antigen are potential epitopes. This has a profound impact on the way we define an appropriate dataset for training and evaluating a B-cell epitope prediction method, and on the limited predictive power of B-cell epitope prediction method (4).

Moreover, in many applications, it is often more important to understand which region of an antigen can be targeted by a specific antibody, or by a group of antibodies, e.g., a library or an antibody repertoire obtained via Rep-Seq (8). This important observation has led to alternative and more well-defined approaches being proposed to address B-cell epitope prediction, where one seeks to predict the cognate target of a given antibody (9, 10). This task is however very complex. First and foremost, the data currently available to perform the task are very scarce. Detailed information on the molecular interactions between an antibody and its cognate antigen target is currently available only from protein 3D structures of antibodies co-crystallized with their target antigen, and currently the protein databank (PDB) only contains ~600 of such antibody-antigen (Ab-Ag) structures. This scarceness makes it extremely complicated to learn rules of Ab-Ag interactions. Moreover, for these rules to be of practical use, we need the 3D structures of the antibodies and antigens that we are investigating, that are in most cases not available. One can potentially predict such structures with well-established methods (11–14), but even then it will be essential to address the effect that the accuracy of these models will have on the performance of any epitope prediction method. As a practical example, protein-protein molecular docking methods can potentially be used, if both the antibody and the antigen structure are available, and usually they demonstrate an accuracy that is slightly superior to antigen-only prediction methods (15, 16). However, the accuracy of docking procedures decreases drastically when applied to structural models of the interacting partners, especially if no additional information on the binding site is available (17–19).

To offer an alternative solution to these problems, here we describe an approach that exploits coarse-grained geometric and physicochemical descriptors of the interacting partners using a combined statistical and Machine Learning approach. We seek to identify, in a set of known structures of antibody-antigen protein complexes, a number of structural, geometric, and physicochemical features that show statistical correlation in interacting paratope-epitope pairs. Next, based on such subset of structural features, we define an algorithm to generate surface patches on a given antigen. We then develop a neural network to discriminate the cognate antibody target (the epitope) from other geometrically similar surface patches. We investigate if including antibody-derived features in the training leads to a better accuracy in the prediction of the correct epitope, and improves the identification of the correct target for a given antibody from a pool of antigens, and the correct target of a given antigen from a pool of antibody targets.

## Materials and Methods

### Structural Dataset

Solved 3D structures of antibodies in complex with their cognate antigen were obtained from IEDB-3D database using the search criteria “Receptor-type: BCR heavy-light” (20, 21). These results were combined with all the unpublished antibody-antigen structures deposited in the PDB, identified using the antibody specific Hidden Markov Models from LYRA (14). Entries of antibodies interacting with other antibodies or T cell receptors were excluded, as were entries where the size of the antigen target was <60 amino acids and entries with a resolution worse than 3 Å. This resulted in 857 antibody-antigen complexes. For each structure, the biological unit was used to make sure we had the functional form of the antigen.

To train and evaluate models without overfitting and over-evaluating predictive power, we partitioned the structures using the tool UCLUST (22) on both the antibody and the antigen sequence. For the antibody clustering, by setting a threshold of 90% sequence identity, we obtained 335 antibody clusters. For the antigen clustering, we obtained 264 cluster using a similarity threshold of 70% sequence identity. These antibody and antigen clusters were combined in a way that all structures with either more than 90% antibody sequence identity or 70% antigen sequence identity were in the same clusters. We obtained 202 antigen-antibody clusters.

A total of 6 merged clusters, consisting of 6 antigen clusters and 8 antibodies clusters, were left out as a final, independent test set (Table S1). The remaining 196 clusters were randomly split into 5 partitions to be used for cross-validation (Figure S1). In each partition, we selected a single representative per antibody cluster, but we retained all complexes in the same antigen cluster. In this way, in our training data we have complexes of the same antigen with different antibody, binding to the same and to different epitopes.

We then defined the binding residues of both paratope and epitope as all exposed residues having a distance between any heavy atoms shorter than 4 Å to any residues in the antigen and antibody, respectively. Exposed residues are defined as those containing at least one heavy atom with more than 2.5 Å^2^ exposed surface area, calculated using PyMOL's python API (23). We should underline that the paratope residues were defined on the actual structure and were not predicted. All data and information about the data and partitioning used in this study can be obtained at http://www.cbs.dtu.dk/suppl/immunology/ASE_Pred/.

### Features

We calculated a number of features on the complexes in our training data set, that were then used to generate putative epitope patches, and to train the algorithm. The complete set of features is described in Table 1.

**Table 1:** Description of the feature used to describe patches.

**Feature**	**Size**	**Description**	**Model**
Amino acid composition	20	Frequency of given amino acid type in patch	Both
Exposed Donors/Acceptors	2	Amount of exposed donor/acceptor atoms	Both
Hydrophobicity score	1	Amount of exposed carbon atoms with distance >2.5 Å to an exposed donor/acceptor atom	Both
Aromatic/Positive/Negative residues	3	Amount of aromatic and positively and negatively charged residues	Both
Principal Components	3	Principal components calculated on x, y, z coordinates.	Both
Size	1	Number of residues within the patch	Both
Patch density	1	Average number of neighbors in patch	Both
RSA max, min & mean	3	Maximum, minimum and average RSA of patch residues.	Antigen
Structural Conjoint Triads	196	Structural conjoint triads based on neighboring residues on surface.	Both
Zernike Moments	7	4th order Zernike Moments excluding 0th and 1st.	Both

*The first four rows (gray) are physio-chemical features, applied in all models. The following three rows (white) are simple structural features also applied in all models. The last three rows (light gray) are more complex structural features only used in the Antigen and the Full model*.

**Figure 1:**
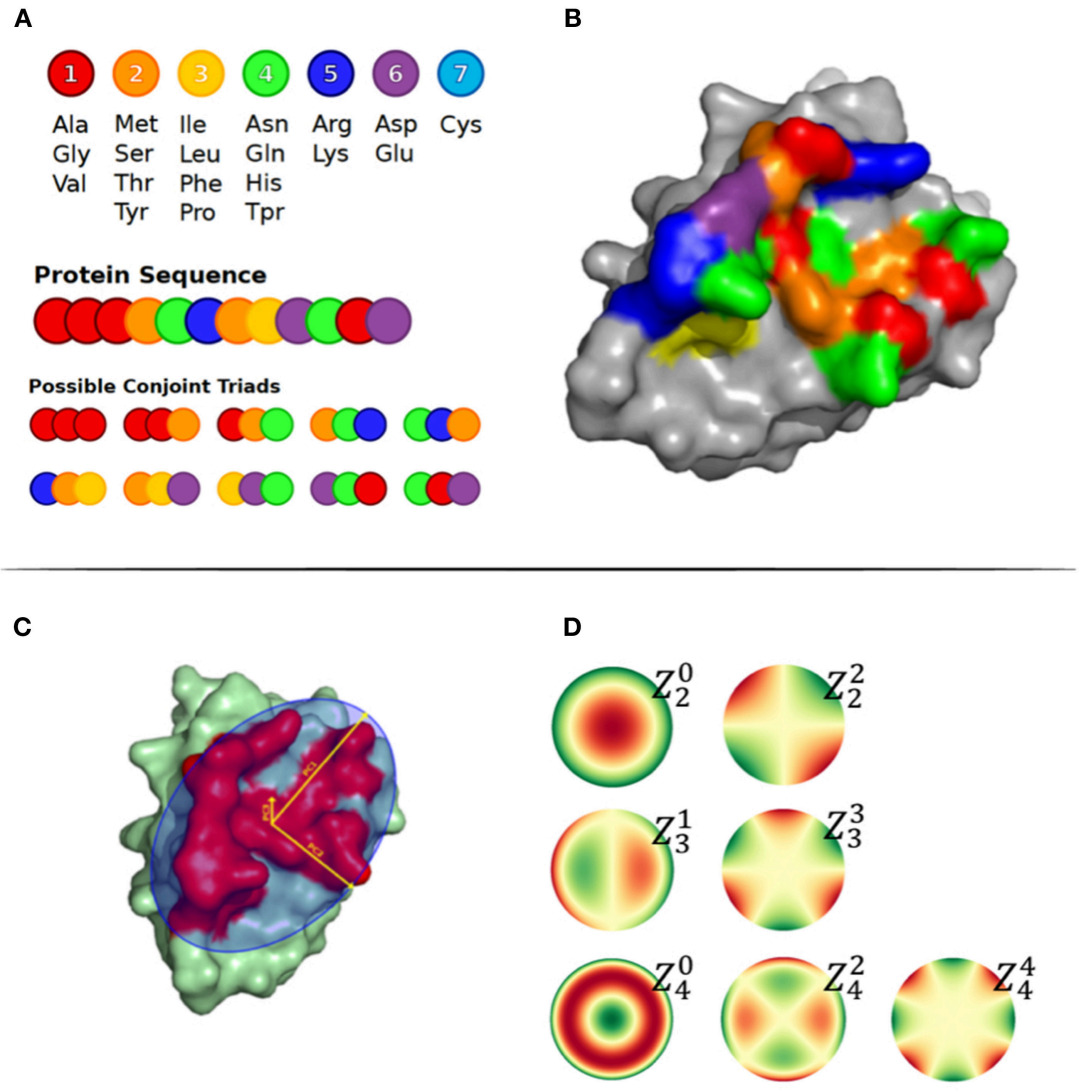
**(A)** Conjoint Triads amino acid classes and representation of method on a sequence level. **(B)** Structural representation of Conjoint Triads classes mapped to an epitope patch. **(C)** The three principal components illustrated on an epitope patch. **(D)** Illustration of 4th order of Zernike Moments' descriptive shape excluding order 0 and 1.

The principal components of the Cα atom coordinates, Zernike moments and other features were used to geometrically represent the surface patches. First, we calculated the three principal components, namely PC1, PC2, and PC3, of the Cα atom coordinates of all residues in a patch, as illustrated in Figure 1C. The size of a patch was defined as the number of residues included in the patch and the patch density was calculated by the average number of neighbor residues for each residue in the patch. The neighboring residues were defined as exposed residues within a 6 Å radius. The max, min, and average Relative Solvent Accessibility (RSA) were calculated using PyMOL as the maximum, minimum and average relative surface area of the residues in the patch. Finally, to describe the structural shape of a patch, we represented each patch as a series of 3D Zernike Moments, that have earlier been used to compare surfaces of proteins and also for antibody binding site classification (24). The Zernike Moments, being translation, scale and rotation invariant, provide a detailed yet robust representation of a surface. In short, the Zernike moments give a compact description of an image by deconvoluting it into a set of primitive functions centered in the middle of the image, each describing a different type of shape. The Zernike Moments were calculated using a modified version of the python package by Scott Grandison et al. (25), where all the atoms in the convex hull region of the patch are used. Examples of the shape description of each moment are shown in Figure 1D. To optimize the computational time and focus on a coarse description of the patch, less dependent on the correct side chain positioning in the structure, only Zernike Moments of order 3 and 4 mainly describing the vertical and horizontal tilts were included.

The amino acid composition and the conjoint triads were used to describe the patch composition statistics. The amino acid composition was calculated as the frequency of each amino acid type in the patch. The conjoint triads are based on structural neighbors (illustrated in Figures 1A,B) (26). Amino acids were assigned to one out of 7 classes displayed in Figure 1A. For a residue X in the patch, all possible triad combinations N_1_XN_2_, where N_1_ and N_2_ are residues that are structural neighbors of X, were identified and labeled as the tuple [C(N_1_), C(X), C(N_2_)], where C() is the class of a residue. Finally, the frequencies of the total 196 non-directional triad types were computed.

Finally, a few features were included describing the physicochemical characteristics of the patch. The exposed acceptor and donor atoms were calculated as the number of exposed h-bond acceptors and donor atoms, respectively, regardless of their actual involvement in any h-bond in the original structure. The hydrophobicity score was calculated by counting the number of exposed carbon atoms with a distance to any exposed acceptor/donor atom of more than 2.5 Å. Additionally, the number of positively and negatively charged residues and number of aromatic residues were included.

### Generating Surface Patches

Surface patches were generated using a Monte Carlo (MC) approach (27). The algorithm starts from a random surface exposed residue (at least one atom exposed more than >2.5 Å^2^), and each MC move can either remove a residue from the current patch, add a residue among the patch's neighboring surface residues, or swap a patch residue with a neighbor one. The moves are then accepted or rejected using the Metropolis criterion.

$$\begin{array}{l} P=min\left(e^{-\Delta E/T},1\right) \end{array}$$

where P is the probability to accept the move, T is a scaling factor that is set to 20 at the beginning of the simulation and reduced over 500 iterations by a factor of 0.985 in each MC step ending at *T* = 0.01045, and E is the following energy function, calculated over a set of features described later:

$$\begin{array}{l} E=\underset{X_{i}\in F}{\sum }{\left(\frac{X_{i}-\bar{X_{i}}}{\sigma_{i}}\right)}^{2} \end{array}$$

where *X*_*i*_ is a feature from the set of features F, and *X*_*i*_, σ_*i*_ are its mean and standard deviation, respectively. Here, the set of features F includes the three ratios between the three principal components, PC1, PC2, and PC3 of the patch and the paratope, the ratio of the size of the patch to the size of the paratope, the ratio between the summed residue surface area of the patch and the surface of the paratope, and the ratio between paratope and epitope patch density.

The mean and standard deviation values of each feature were determined from the actual epitope-paratope pairs in a cross-validated manner, so that the patches generated for any antigen in a given partition are constructed from values obtained from the remaining 4 partitions. Using this MC approach with a total of 500 MC moves per simulation, 300 patches (MC patches) were generated per antigen.

### Training Set

In order to develop a function for scoring putative epitope/paratope patches, we first defined a training set composed of real and MC generated epitope-paratope pairs. Target values were assigned to MC generated epitope-paratope pairs based on their overlap with the real pairs as the product of the precision (proportion of residues in the patch that are part of the actual epitope) and recall (proportion of epitope residues included in the patch). This target value is hence 1 if the patch overlaps perfectly with the actual epitope, and zero if no overlap is present. To evaluate how well a model predicts patches overlapping to the real epitope, we defined patches with a target value above 0.25 as a highly overlapping (HO) patch.

We included the actual paratope-epitope pair, together with up to 10 non-redundant epitope-overlapping MC patches (target value >0.0075) from each complex in the training set. These were selected using a Hobohm1 (28) like approach by sorting the patches based on their target value and iteratively including only patches with <60% overlap in residues to patches previously included. Similarly, up to 50 non-redundant MC patches with target value ≤0.0075 were added, with the difference of not being sorted on their target value.

Moreover, for each complex, we included 10 mis-paired paratope-epitope patches, obtained by pairing the real epitope patch with the paratope of an antibody from a different antibody cluster. Given the very high specificity of antibodies, we assumed that they do not bind a random antigen, and therefore assigned a target score of 0 to the mis-paired patches.

### Neural Network Architecture and Training

A Feed Forward Neural Networks (FFNN) model was constructed using the python package Keras (29), with two hidden layers each having 25 neurons, sigmoid activation function at all neurons and ADAM as the optimizing function. Three models were made (Full, Minimal and Antigen model) using different features to encode the patches. Table 1 shows a summary of which features were used in the different models. The Full model included all calculated features, i.e., one data point consists of 471 features, where 234 describe the paratope and 237 describe the antigen patch. The Minimal model did not include the last three feature sets resulting in 62 features, 31 for each antibody and antigen patch. The Antigen model was similar to the Full model, however, only including the 237 antigen features.

Feed forward neural networks were trained and their performance were evaluated using a nested 5 partition 10-fold cross validation: one of the 5 partitions was in turn left out from the model training, and then the remaining 4 partitions were next split into 10 random sub-partitions maintaining the original clustering, and models were trained using 10-fold cross-validation with early stopping. Finally, the ensemble of these 10 models was used to predict the left-out partition in the outer 5-fold cross validation.

## Results

**Figure 2:**
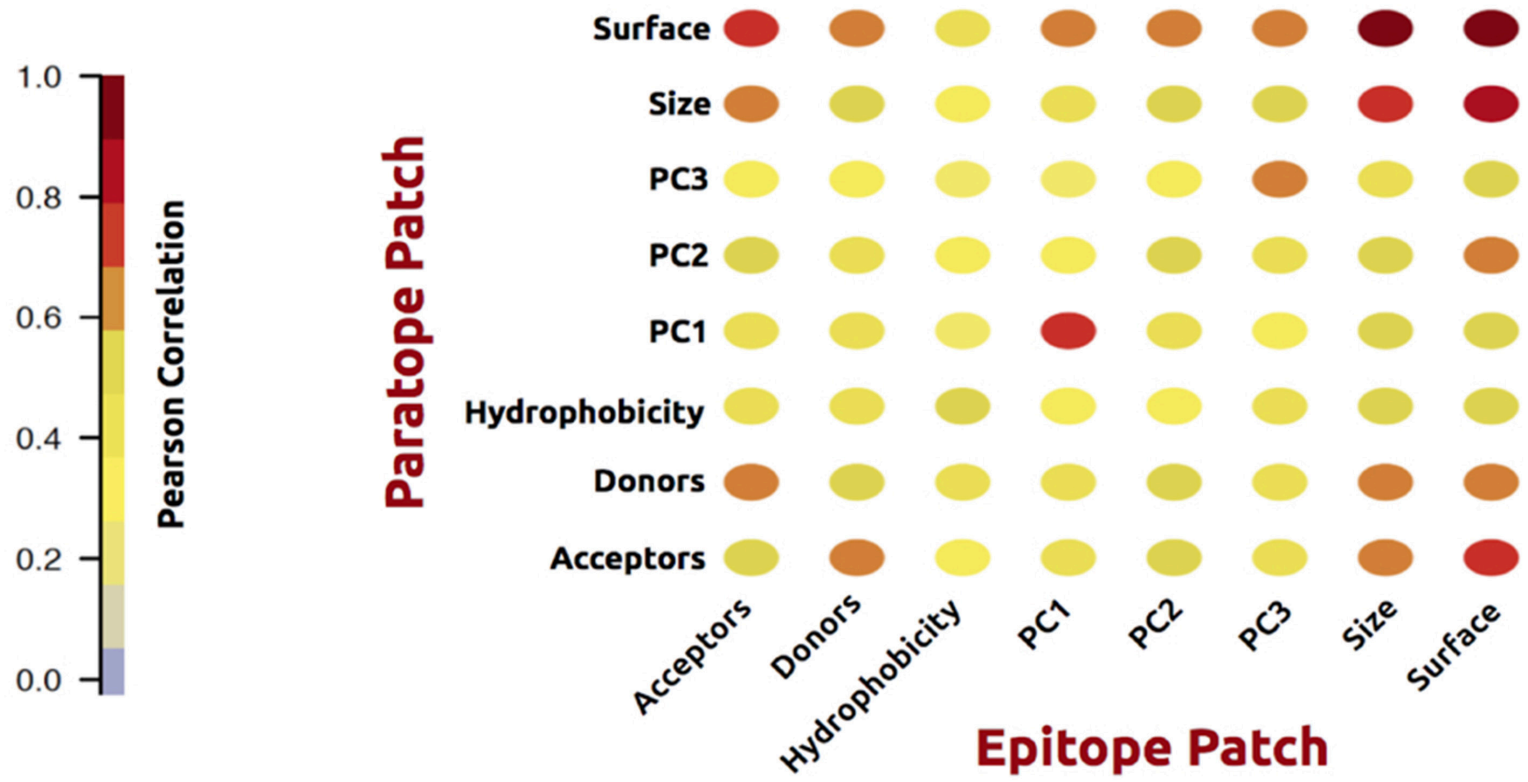
Correlation matrix of structural and physicochemical features of the true paired paratope and epitope patches.

As an initial analysis, we investigated correlations between structural and physicochemical properties of actual paratope and epitope patches. We compared the correlations of various structural features (PC1-3, size, and surface) measured on both the paratope and the epitope patch, as shown in Figure 2.

As expected, these analyses demonstrated a high correlation between corresponding structural properties of the paratope and epitope; i.e., the size of the epitope is to a high degree predictable from the paratope size. The same holds for the epitope shape (PCs) and surface. Similar results were obtained for physicochemical features: hydrophobicity, h-bond acceptors and donors. Unsurprisingly, the number of available acceptors in a patch correlates with the number of available donors in the corresponding partner patch. These observations overall suggest that information contained within the antibody is of potentially use to gain insight into the shape and physicochemical properties of the cognate epitope.

In order to prove that the observed correlations could be used to generate an improved prediction, we tested different prediction models each trained and evaluated using nested cross-validation on the training data (for detail on model architecture, training hyper-parameters and model features see methods). We trained 3 such methods using different subsets of the features from each patch. The first model was trained on the **Antigen features only** (i.e., geometrical properties of the antigen patch combined with amino acid composition, amount of exposed Donor/Acceptor atoms, hydrophobicity, and amount of Aromatic/Positive/Negative residues). Here, no information on the paratope patch was included. The second model was a **Minimal model**, which was trained using a minimal set of features of antigen and paratope patches. The third was the **Full model** expanding the Minimal model to include the additional features of structural conjoint triads, Zernike Moments, and maximal, minimal and average relative surface exposure of the paratope and epitope patches (for details see materials and methods).

### Epitope Ranking

We evaluated the performance of each of the three models by scoring, for each structure in the independent test partition, the actual epitope and 300 MC patches. The 301 patches were sorted according to their score, and the performance was reported as the relative number of MC patches with a score higher than the epitope (F_rank_). In this way, a perfect prediction where the epitope is ranked at the top of the list would get a F_rank_ score of 0, and a F_rank_ score of 0.5 would correspond to a random prediction (30, 31).

**Figure 3:**
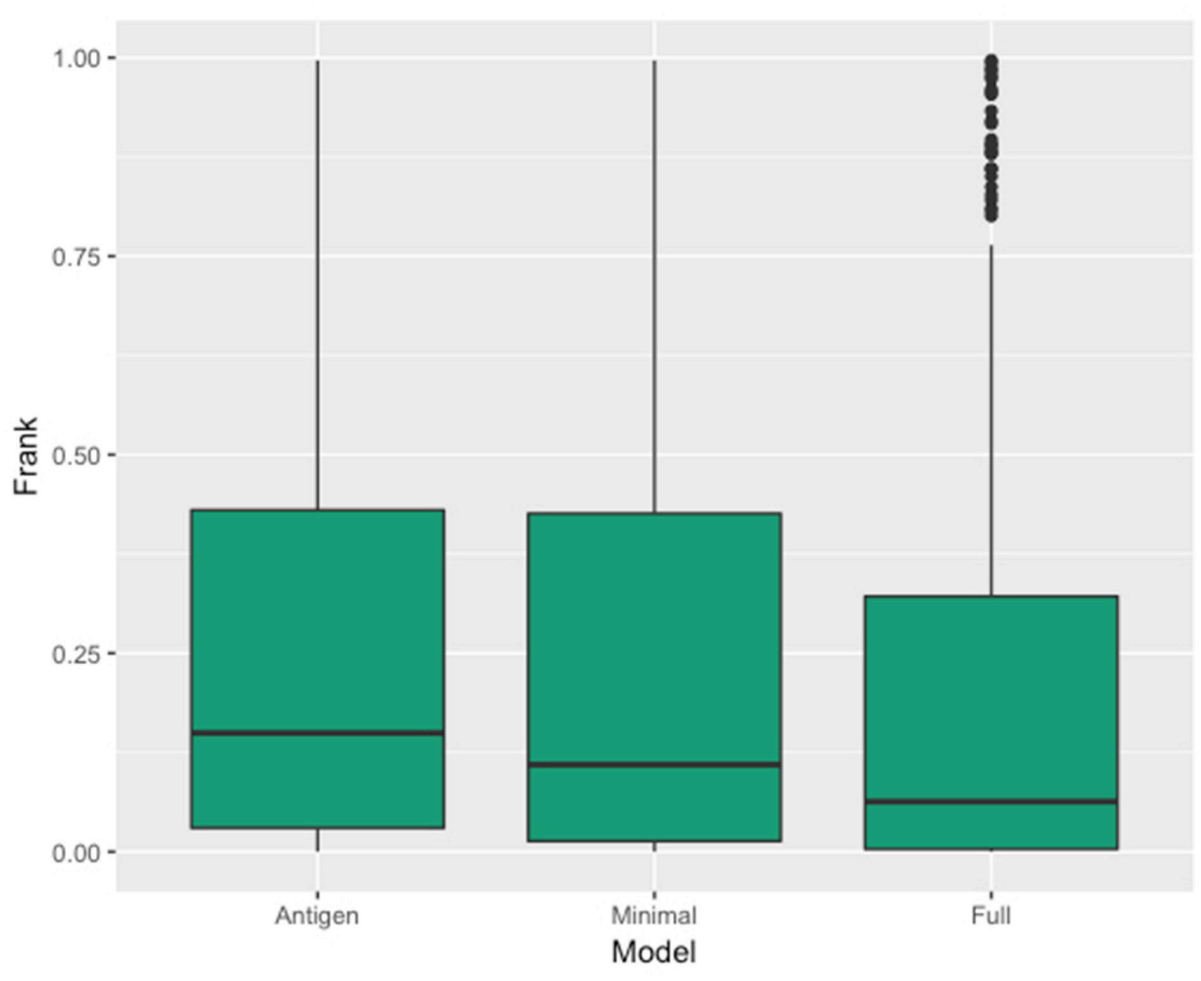
Box plot showing the distribution of the real epitope ranks within each Antibody-Antigen structure for the three prediction models; Antigen, Minimal, and Full.

In Figure 3, we show the results of this analysis for the 3 models in terms of a boxplots of the F_rank_ values for each of the three prediction. These plots confirm the superior performance of the Full model compared to the other two models with a median rank performance of 7.4% compared to performance values of 10.9 and 15% for the minimal and antigen models respectively. The results demonstrate that incorporating information of the antibody in the prediction model results in a high gain in predictive power.

**Figure 4:**
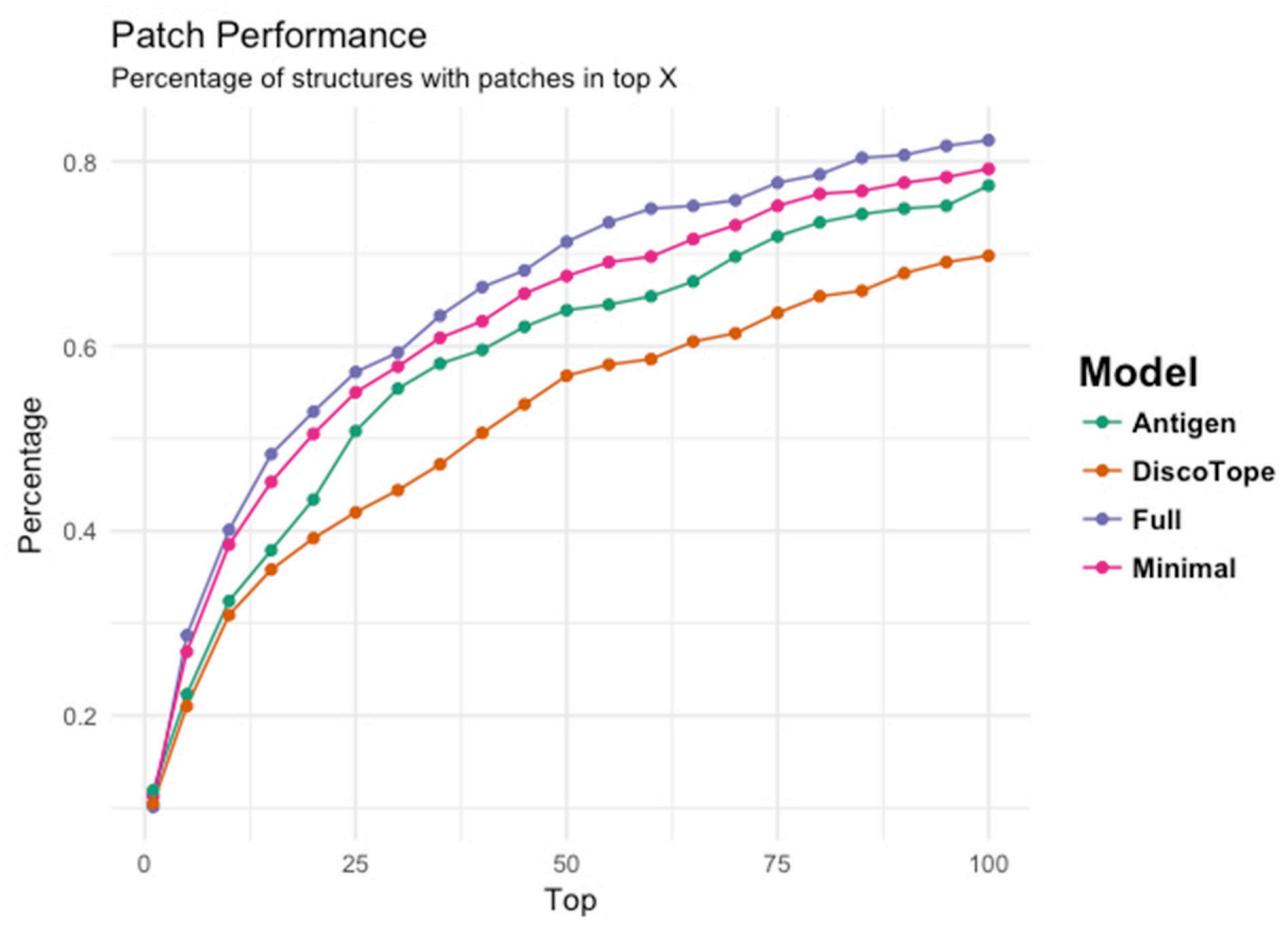
The ability of the four models' (Antigen: green, Minimal: pink, Full: purple, and DiscoTope-2.0: orange) to identify high overlapping patches. X-axis indicating number of top predicted patches included and Y-axis showing the percentage of structures having at least one high overlapping within the selected pool.

Figure 4 provides another way to illustrate the predictive performance of the different models. Here, we show how many of the structures have a highly overlapping patch within the top 1, 5, 10, 15, 20 up to top 100 MC patches for each of the 3 models. Additionally, we compared the performance of each models to DiscoTope-2.0. Here, the patch score was calculated as the sum of DiscoTope predictions over all residues in a given patch. This analysis again clearly demonstrates that the Full model has the highest performance of all models confirming that the predictive power is increased by integrating information from the cognate antibody. It is also interesting to observe that even the Antigen model achieves results that are slightly improved compared to Discotope-2.0.

### Antibody-Antigen Pairing

One of the main goals of an antibody-guided epitope prediction tool would be to identify the cognate antigen target of a given antibody from a pool of potential antigens. To benchmark the tool in such a setting, we scored each “true” epitope patch against all paratope patches (from antibodies with different epitopes) within each data partition and registered the rank of the true paratope in this sorted list (Antibody Rank). This benchmark resulted in average and median ranks of 23.7 and 15.6%, respectively. Further details on the different performance measures used are given in Table 2. Next, for each antibody, we similarly score the paratope against the epitopes from all structures within the given data partition, and identified the rank of the cognate epitope patch within this sorted list (Antigen Rank), resulting in an average rank of 24.8% and a median rank of 17.5%. These analyses demonstrate that the model is capable of differentiating between real and mis-paired epitope-paratope pairs.

**Table 2:** Description of the ranking measurements used to describe performance of the models.

**Measurement**	**Description**
Epitope Rank	The F_rank_ of the real epitope patch against the ~300 Monte Carlo patches.
Antigen Rank	The F_rank_ of the cognate epitope patch toward a given paratope against a pool of epitope patches from other antigens.
Antibody Rank	The F_rank_ of the cognate paratope patch toward a given epitope against a pool of paratopes from other antibodies.
Structurally Similar Antibody Rank	The F_rank_ of the cognate paratope patch toward a given epitope against a pool of paratopes from other antibodies with structurally similar paratopes.
Monte Carlo Antibody Rank	The F_rank_ of the cognate paratope patch toward a given antigen against a pool of paratopes from antibodies with structurally similar paratopes. The paratope score is defined by the average of top 5 scoring Monte Carlo patches.
Monte Carlo Antigen Rank	The F_rank_ of the cognate epitope of an antigen toward a given paratope against a pool of epitopes from other antigens with structurally similar paratopes. The antigens score is defined by the average of top 5 scoring Monte Carlo patches.
First HO Patch	The F_rank_ of the highest predicted patch highly overlapping the real epitope (target value above 0.25) in the list of predicted antigen patches sorted by prediction value.

As the PCs are highly correlated between paratope and epitope patches, one could speculate that the above performance values were driven by these structural similarities. To investigate this, we repeated the experiment only including paratopes of similar shape. We performed a K-means clustering (with 5 clusters) within each test partition, based on a vector of the three PCs of the paratope. We then recalculated the ranking of each paratope only against paratopes in the same cluster, hence with similar PCs (Structurally Similar Antibody Rank). This resulted in an average Structurally Similar Antibody Rank of 25.5% (compared to the average Antibody Rank of 23.7% obtained when comparing to all paratopes). This result indicates that the geometric differences (i.e., PCs) are not the main drivers of the predictive performance of the model.

In the benchmark calculations conducted so far, we have focused on identifying surface patches corresponding to or overlapping the cognate epitope of a given antibody. Another important application of the proposed method would be to identify the cognate antibody target of an antigen without involving information about the actual epitope. To assess to what degree this was possible, we scored the 300 MC patches of an antigen against all paratopes with similar structural properties to the true paratope (i.e., within the PC cluster of the true paratope) and assigned a score to each paratope from the top 5 MC patch predictions. The paratopes were then sorted based on this score and the rank of the true paratope reported (Monte Carlo Antibody Rank). Doing this, resulted in an average Monte Carlo Antibody Rank of 31.2%.

Finally, and with the intent of benchmarking the performance of the model for selecting the correct antigen target given an antibody, for each antibody we predicted its pairing to all antigens with similar structural epitope properties (i.e., within the same epitope PC cluster). We did this by scoring all 300 MC patches of a given antigen to the given paratope patch, and assigning a single score as the average of the top 5 MC patch predictions. All antigens were then sorted by this score, and the rank of the true antigen was measured (Monte Carlo Antigen Rank). This resulted in an average Monte Carlo Antigen Rank of 29.9%, showing similar performance as the antibody selection.

While these latter results are not striking, they demonstrate how, using the proposed framework, we are able to construct models that are capable to a high degree of identifying both the correct antibody target for a given antigen, and the correct antigen target of a given antibody.

### Independent Test Set

Finally, the model was evaluated on the independent test data set consisting of eight structures non-redundant to the training data. We generated 300 MC patches for each antigen and the rank of the highest scored HO patch in this set of patches was recorded (**First HO Rank**). Next, the true epitope was included and ranked (**Epitope Patch Rank**). Additionally, we evaluated the ability of each tool of identifying the correct antigen (**Antigen rank**) given the paratope and the correct antibody (**Antibody Rank**) given the epitope.

This benchmark was performed on the Full model, on Discotope-2.0, and on ClusPro (16, 32), an antibody-specific docking protocol. Again for DiscoTope, the patch score was calculated as the sum of the DiscoTope score for all residues in the given patch. For ClusPro, as recommended, we used the cluster size as patch score. For the Antigen and Antibody ranks, as before, the test was conducted by ranking the score of the true epitope patch to the score of the epitope patches from the other 7 antigens in the benchmark when paired with the actual paratope, and by ranking the true paratope patch to the paratopes of the other 7 antibodies when paired with the actual epitope. The results of the First HO rank and Epitope Patch rank comparison between the Antigen model, the Full model and Discotope-2.0 are included in Table 3, and demonstrate a consistently improved performance of the Full model. We observe that the rank of the first high overlapping patch (First HO rank) in more than half of the cases (175) is lower than the rank of the true epitope. This suggests both that the Monte Carlo generation algorithm is in more cases capable of generating patches that structurally overlap the true epitope, but also that there is room for improvements in the prediction model (the actual epitope should ideally be ranked at the top). For our models, we always generate a total amount of 300 MC patches, but ClusPro generates a variable number of solutions, usually between 20 and 30. To perform a fair comparison against ClusPro, we reduce the MC patches to ~30 patches per structure by using a Hobohm 1 approach, sorting the patches in a given structure by prediction value and excluding any patches sharing 35% or more of its residues with any other patch higher up on the list (Table 4). We observe that, though the Full model and ClusPro have a similar ability to identify HO patches, ClusPro has almost no predictive power in pairing the right epitope and paratope together (Antigen and Antibody ranks values are close to 50%).

**Table 3:** Benchmark of 8 antibody-antigen PDB structures non-redundant to training, comparing DiscoTope-2.0, the Antigen Model and the Full Model developed here.

**PDB ID**	**DiscoTope-2.0**		**Antigen model**		**Full model**	
	**Epitope patch rank (%)**	**First HO rank(%)**	**Epitope patch rank(%)**	**First HO rank(%)**	**Epitope patch rank(%)**	**First HO rank(%)**
3RKD	95.3	44.7	60.1	0	72.7	0.3
4EDW	52.2	53	39.2	19	10.6	25.3
5B3J	3.3	22	31.8	56.3	0.3	17.3
5DHV	92	51	75	12.6	7.6	8.3
5SY8	5.3	0.3	53.4	0.3	22.2	0
5TZ2	72.8	50.3	11.6	10.6	2.6	9.6
5TZT	0	39.3	19.9	5.0	87.3	20.0
5TZU	54.2	43.7	20.2	0.6	2.6	0.3
AVERAGE	38	46.9	39	13.1	25.8	10.3
MEDIAN	44.2	53.2	35.5	7.8	9.1	9.1

*Epitope Patch Rank indicates how well each model ranks the real epitope patch within the 300 Monte Carlo patches. First HO patch shows how high the first high overlapping patch (target value > 0.25) ranks within the set of 300 MC patches. A rank of 0% means it was ranked highest and a rank of 100% is ranked lowest or that no HO patch exists for the structure*.

**Table 4:** Benchmark of 8 antibody-antigen PDB structures non-redundant to training, comparing DiscoTope-2.0, ClusPro and the Full Model developed here.

**PDB ID**	**DiscoTope-2.0**	**Full Model**			**ClusPro**		
	**First HO rank(%)**	**First HO rank(%)**	**Antigen rank(%)**	**Antibody rank(%)**	**First HO rank(%)**	**Antigen rank(%)**	**Antibody rank(%)**
3RKD	86.6	4.5	87.5	25	0	75	75
4EDW	79.1	100	37.5	37.5	19	25	12.5
5B3J	59.8	22.3	0	0	43.3	87.5	50
5DHV	33.3	37.5	12.5	0	100	50	62.5
5SY8	40	0	12.5	12.5	23.3	37.5	87.5
5TZ2	92.3	21.4	0	0	0	50	75
5TZT	32.4	30	62.5	37.5	100	37.5	62.5
5TZU	86.3	4.3	12.5	0	0	0	0
AVERAGE	63.8	27.5	28.1	14.1	35.7	45.3	53.1
MEDIAN	69.5	21.9	12.5	6.2	21.2	43.8	62.5

*First HO patch shows how high the first high overlapping patch (target value > 0.25) ranks within the set of non-redundant MC patches. Antigen and Antibody ranking show how well the Model can select the correct antigen given a paratope and select the correct antibody given an epitope, respectively. Patch scores for the Discotope-2.0 were calculated as described in the text. A rank of 0% means it was ranked highest and a rank of 100% is ranked lowest or that no HO patch exists for the structure*.

## Discussion

Prediction of B cell epitopes has proven extremely challenging. One reason for this is the ill posed question most often put forward when seeking to predict B cell epitopes, where the aim is to predict whether a given surface patch of an antigen is a potential epitope, i.e., if one or more of the many billion antibodies potentially present in a host can target this region. In this current study, we formulate a more precise question, and ask if we can predict the epitope target of a given cognate antibody. The answer to this question is yes, as results of this study illustrates the substantial gain of including information on the cognate antibody for specific epitope predictions. Using 3D structures of antibody-antigen complexes, our analyses identified multiple structural and physicochemical features that correlate between interacting paratope and epitope patches. By using these features in a Feed Forward Neural Network, we demonstrated that even simple features extracted from cognate antibody improved the accuracy in predicting specific epitopes. Additionally, we showed that more complex features, such as Zernike Moments, could further improve the predictive power of the model. Comparing to DiscoTope-2.0, a structural B-cell epitope predictor that only uses the antigen structure, we showed both in cross-validation and on an independent dataset of structures, that our model performed better in identifying patches overlapping with real epitope patch. Additionally, the model showed promising results in pairing a given antibody to its cognate antigen from a pool of antigens and vice versa. When compared to ClusPro, an antibody-specific docking protocol, we observed that our model, but not DiscoTope, had a similar predictive power to identify the epitopes. Additionally, using ClusPro to select the correct antibody-antigen pairs, we obtain results close to random, whereas our model could to a fair degree perform this task correctly. These results indicate the that our model has not only to a high degree learned to predict the correct epitope, but also to pair it with the correct antibody.

Despite the very promising results of our model, it is clear that the predictions are far from perfect. This in particular true when it comes to identifying the correct antigen target for a given antibody and vice versa the correct antibody target for a given antigen. On a repertoire-scale, where more than 10^6^ antibodies can be isolated, our current results of identifying an antibody for a specific epitope in the top ~31% of hits would still mean testing 3.1^*^10^5^ antibodies, which cannot realistically be tested for specificity using conventional experimental approaches. Thus, significant further improvements in prediction performance are necessary to enable such broad applications. However, for many applications, the antibody repertoire that needs to be considered can be narrowed by additional information, such as in the case of vaccine induced plasmablasts, and the identification of clusters in antibody sequences that are expected to have related specificity would allow to limit sequences to be considered to a set of cluster representatives. We expect that going forward there will be an iterative improvement of both experimental and computational approaches to address these challenging questions as there has been for predictions of T cell epitopes, and have here confirmed the feasibility of improving our ability to predict B cell epitopes by developing antibody specific prediction models.

One important explanation for the relative poor predictive model of our model is lack of training data. By downsampling our training data, we can show (data not shown) that the amount of high quality data (i.e., 3D structural data) is still too limited, and that the performance of the method improves significantly as more structures are included in its training. Also, we often observe that the actual epitope is not scored in the very top of the prediction list, when compared to MC patches. Adding more data and more features to the model might lead to future improvements.

A second caveat is that the current results, though focusing on coarse-grained descriptors, have been generated on solved structures rather than on models. Kilambi and colleagues have investigated how docking methods can be used to discriminate between cognate and non-cognate antibody-antigen pairs (33). Analyzing the native-bound structure of 17 antibody-antigens complexes, they could demonstrate a very high performance, and in 80% of the cases rank the cognate antibody as the top target for a given antibody. However, when using modeled antibodies structures in the benchmark that number went down to 12%. In our work, we likewise benchmark the model performance using the native structure of the bound antigen-antibody complexes, and even though the modeling framework used here is more coarse-grained compared to that underlying protein docking, we would still expect some drop in performance when applying the framework to unbound or modeled antibody and antigen structures.

Also, the current approach depends on the structural properties of the antibody paratope, that are used when generating epitope-like patches on the surface of the antigens. Given this, we expect the performance to be affected when using predicted paratope patches. Nevertheless, in the last few years a plethora of methods for antibody structure and paratope prediction have been developed, in most cases achieving extremely high accuracy (34–36).

Even with the aforementioned limitations, we believe that our approach, that uses a statistical and machine learning approach to successfully include antibody information in B-cell epitope prediction, can be of great importance in many applications, such as *in silico* antibody library screening, identification of antibody targets for vaccine development and immunotherapy, and analysis of antibody cross-reactivity.

## Author Contributions

MJ: prediction model, data generation, analyses and paper writing; SM: data generation, proof reading; BP: project development, guidance, proof reading and writing; MN: project development, guidance, proof reading and writing; PM: project development, guidance, proof reading and writing.

### Conflict of Interest Statement

The authors declare that the research was conducted in the absence of any commercial or financial relationships that could be construed as a potential conflict of interest.
