# Supplementary material for: Antibody Specific B-Cell Epitope Predictions: Leveraging Information From Antibody-Antigen Protein Complexes
Source: Front Immunol. 2019 Feb 26;10:298. doi: 10.3389/fimmu.2019.00298 (PMC6399414; doi:10.3389/fimmu.2019.00298)
Supplement: Supplementary file 1 [file Table_1.DOCX]

**Figure S1:** **A:** Heat map showing combined antibody heavy and light chain sequence similarity for

all the 857 structures. **B:** Heat map showing antigen sequence similarity for 857 structures. When

comparing multi-chain antigens, only the chains with the highest sequence similarity are reported.

| ***PDB ID*** | ***Antibody***  **Species** | ***Heavy Chain*** | ***Light Chain*** | ***Antigen Species*** | ***Name*** | ***Chains*** |
| --- | --- | --- | --- | --- | --- | --- |
| *3RKD* | N/A | H | L | Hepatitis E Virus E2s domain | Capsid protein | A |
| *4EDW* | N/A | H | L | Homo Sapiens | Beta-nerve growth factor | V |
| *5B3J* | N/A | H | L | Xenopus laevis, Rattus norvegicus | NMDA glutamate receptor subunit, Glutamate receptor ionotropic, NMDA 2B | B,D |
| *5DHV* | N/A | H | L | HIV-1 | Protein Rev | M |
| *5SY8* | N/A | H | L | HIV-1 | 10E8 EPITOPE SCAFFOLD T117v2 | O |
| *5TZ2* | Homo Sapiens | H | L | Homo Sapiens | Leukocyte surface antigen CD47 | C |
| *5TZT* | Homo Sapiens | A | B | Homo Sapiens | Leukocyte surface antigen CD47 | D |
| *5TZU* | Homo Sapiens | H | L | Homo Sapiens | Leukocyte surface antigen CD47 | C |

**Table S1**: External dataset of 8 non-redundant antibodies, with 6 different antigen clusters (5TZT, 5TZU, 5TZ2 same antigen cluster) all non-redundant both to antibodies and antigens of training dataset.
