# Supplementary figures and images for: Antibody Specific B-Cell Epitope Predictions: Leveraging Information From Antibody-Antigen Protein Complexes
Source: Front Immunol. 2019 Feb 26;10:298. doi: 10.3389/fimmu.2019.00298 (PMC6399414; doi:10.3389/fimmu.2019.00298)

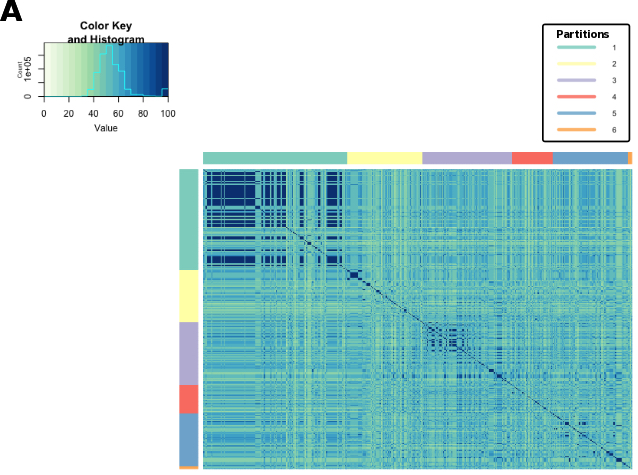

Supplement: Supplementary file 2 [file Image_1.JPEG]

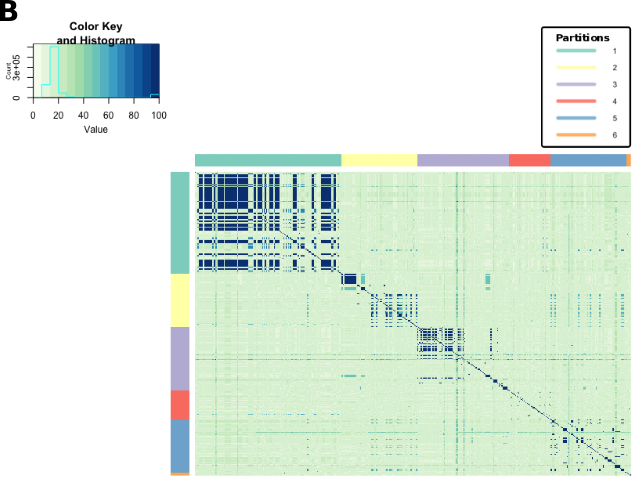

Supplement: Supplementary file 3 [file Image_2.JPEG]
